# Study on Dalfampridine in the treatment of Multiple Sclerosis Mobility Disability: A meta-analysis

**DOI:** 10.1371/journal.pone.0222288

**Published:** 2019-09-12

**Authors:** Jianzhen Shi, Xiaohui Wu, Yanmei Chen

**Affiliations:** 1 School of Science Nantong University, NanTong, P.R. China; 2 Affiliated Hospital of Nantong University, NanTong, P.R. China; 3 School of Environmental and Chemical Engineering Jiangsu University of Science and Technology, Zhenjiang, P.R. China; University of Mississippi Medical Center, UNITED STATES

## Abstract

**Objective:**

Systematic Review was used to evaluate the efficacy and safety of Dalfampridine (DAP) in the treatment of Mobility Disability (MS) in patients with Multiple Sclerosis.

**Methods:**

Clinical randomized controlled studies about DAP and placebo in the treatment of Mobility Disability in patients with Multiple Sclerosis until March 2019 were explored by searching Embase, PubMed, Cochrane, Web of Knowledge, and ClinicalTrials.gov. Literature screening, data extraction, quality assessment, and statistical analysis were performed by using Stata 14.0.

**Results:**

10 papers were included in the meta-analysis, and the number of patients was 2100. In conclusion, the application of DAP in clinical can significantly improve the Mobility Disability of patients [OR = 2.73, 95%CI (1.66, 4.50), P<0.001, I^2^ = 74.1%] and boost the mobility speed of patients in Timing 24 Minute Walk Test (T24FW) [SMD = 3,08, 95%CI(1,58, 4.58), P<0.001, I^2^ = 98.7%]. There are no significant differences of the incidence of adverse events [RR = 1.06, 95%CI (0.99, 1.14), P = 0.928, I^2^ = 0.0%] and urinary tract infection [RR = 1.21, 95%CI(0.91, 1.60), P = 0.145, I^2^ = 37.2%] between the DAP test group (Doses≤10 mg) and the placebo control group, and the incidence of adverse events [RR = 1.14, 95%CI(1.02, 1.28), P = 0.793, I^2^ = 0.0%] and urinary tract infection[RR = 3.05, 95%CI(1.04, 8.99), P = 0.680, I^2^ = 0.0%] for the DAP test group (Doses>10 mg) is a litter higher than the placebo control group.

**Conclusion:**

DAP can effectively improve Mobility Disability in patients with Multiple Sclerosis, which is safe and reliable in specific DAP usage doses.

## Introduction

Multiple Sclerosis (Mobility Disability, MS) is a kind of inflammatory disease, which is immune-mediated by the central nervous system, that can cause the demyelination of axonal to occur nerve impulse conduction block [1]. It affects multiple areas of white matter by forming plaques, and the periventricular is particularly common [2]. According to reports [3], there are more than 2.5 million people with Mobility Disability worldwide and more than 1 million people with Mobility Disability in the United States alone. The clinical manifestations of Mobility Disability are physical disabilities such as Mobility Disability, cognitive and sensory disorders, wherein Mobility Disability are the most common one [4].

Clinical treatments for Mobility Disability include medication and physical therapy [5–6]. Medications therapy usually use anti-inflammatory drugs (such as steroids) to control the acute onset of the disease, use disease-modifying drugs to reduce the frequency and severity of recurrence, and specific drugs are used to control the symptoms associated with MS [6]. Studies show that drug treatment can effectively slow down and control the severity of MS [7]. However, its ability to treat or improve Mobility Disability is inconspicuous. DAP is the first drug approved by the US FDA for the treatment of MS. Clinical trials have shown that it can effectively improve the Mobility Disability of patients [8]. DAP is a potassium channel blocker whose mechanism of action is to block potassium channels by restoring nerve impulse conduction [9]. Blocking of potassium channels can further promote and activate synaptic transmission [10], to delay repolarization of postsynaptic action potentials. Consequently, it will result in the influx of a large amount of Ca^2+^ and increasing the release of acetylcholine [11–12]. And it can also directly activate high voltage-dependent Ca^2+^ channels independent of potassium channels [13]. Investigations validated the force of DAP in the treatment of Mobility Disability in patients with MS. However, evidence-based medicine evidence is slightly inadequate, with no systematic data to explain its safety and effectiveness. Finally, DAP is mainly for patients in western countries. It is hoped that this meta-analysis will have particular reference significance for clinicians in Asia.

## 1 Materials and methods

Data collection and reporting met the statement of Preferred Reporting Items for Systematic Reviews and Meta-Analyses (PRISMA) (S1 Appendix).

### 1.1 Inclusion and exclusion criteria

#### 1.1.1 Type of research

Randomized controlled trial (RCT), the language is limited to English.

#### 1.1.2 Research object

Patients with clinically diagnosed Mobility Disability are not limited in age, gender, and duration of disease.

#### 1.1.3

The test group was treated with DAP, and the control group treated with placebo. Intervention time and dosage are not limited.

#### 1.1.4 Outcome measures

Mobility Disability improvement rate, walking speed change lv, serious adverse event rate, and adverse reactions of urinary tract infection.

#### 1.1.5 Exclusion criteria

1) Observation and retrospective studies; 2) cross-over study; 3) Repeated published research; 4) Conference summary, case analysis, literature review; 5) Non-English literature.

### 1.2 Literature search strategy

Searching databases selected the relevant studies from November 2008 to March 2019 in Embase, PubMed, Cochrane, Web of Knowledge, and ClinicalTrials.gov. The language is limited to English, and the two authors use the combination of subject terms and free words to search for the search terms, as shown below. “Multiple Sclerosis”, “Sclerosis, Multiple”, “Sclerosis, Disseminated”, “Disseminated Sclerosis”, “4-Aminopyridine”, “DAP”, “Pymadine”, “VMI-103”, “Fampridine-SR”, “randomized controlled trial”, For related search strategies, see S1 File.

### 1.3 Literature screening and data extraction

The two authors (Jianzhen Shi and Yanmei Chen) independently screened the literature and cross-checked. If the opinions encountered during the screening process are different, the third author (Xiaohui Wu) will identify according to the exclusion criteria and outcome indicators. When the literature is screened, the title and abstract of the literature are read one by one, and the full text is read to determine whether it is the abstract included is sufficient. The data extraction content includes 1) Basic information for inclusion in the study: Research topics, first author, published magazine. 2) Baseline characteristics of the study subject (Including age, gender, total sample size) and Intervention. 3) Key elements of bias risk assessment (Random method, whether to implement a blind method, Assignment hiding) 4) Outcome indicator of concern and the result measurement data.

### 1.4 Risk assessment of bias in inclusion studies

The Cochrane bias risk assessment tool was used by both authors to assess the risk of bias. The kappa statistic calculated Inter-rater reliability for the study selection.

### 1.5 Statistical analysis

Data analysis was performed using Stata 14.0 and Revman. The second categorical variable uses odds ratio (OR) and risk ratio (RR) as the effect index, the continuous variable uses the standardized mean difference (SMD) as the effect indicator, and each effect indicator gives the point estimate and its 95% CI. The heterogeneity of the included studies was assessed using the Cochran Q statistic test (test level is P = 0.1), and the size of heterogeneity between studies was examined by I^2^ value. If I^2^≥0%, it is non-heterogeneous, I^2^≥25% is considered to be mild heterogeneity, I^2^≥50% is considered to be moderate heterogeneity, and I^2^≥75% is considered to be severe heterogeneity. If there is no statistical heterogeneity between the studies, a fixed-effect model is used for meta-analysis. If there is statistical heterogeneity between the results of each study, a random effect model is used for meta-analysis, and meta-regression analysis was used to explore possible sources of heterogeneity. We used the Funnel plot and Egger test to evaluate the publication bias of the meta-analysis, which P <0.05 indicates significant publication bias. Moreover, sensitivity analysis is used to confirm that weather any research cases caused some inadequacy influences in the meta-analysis of fixed/random effects by using STATA software.

## 2 Results

### 2.1 Document screening process and results

**Fig 1:**
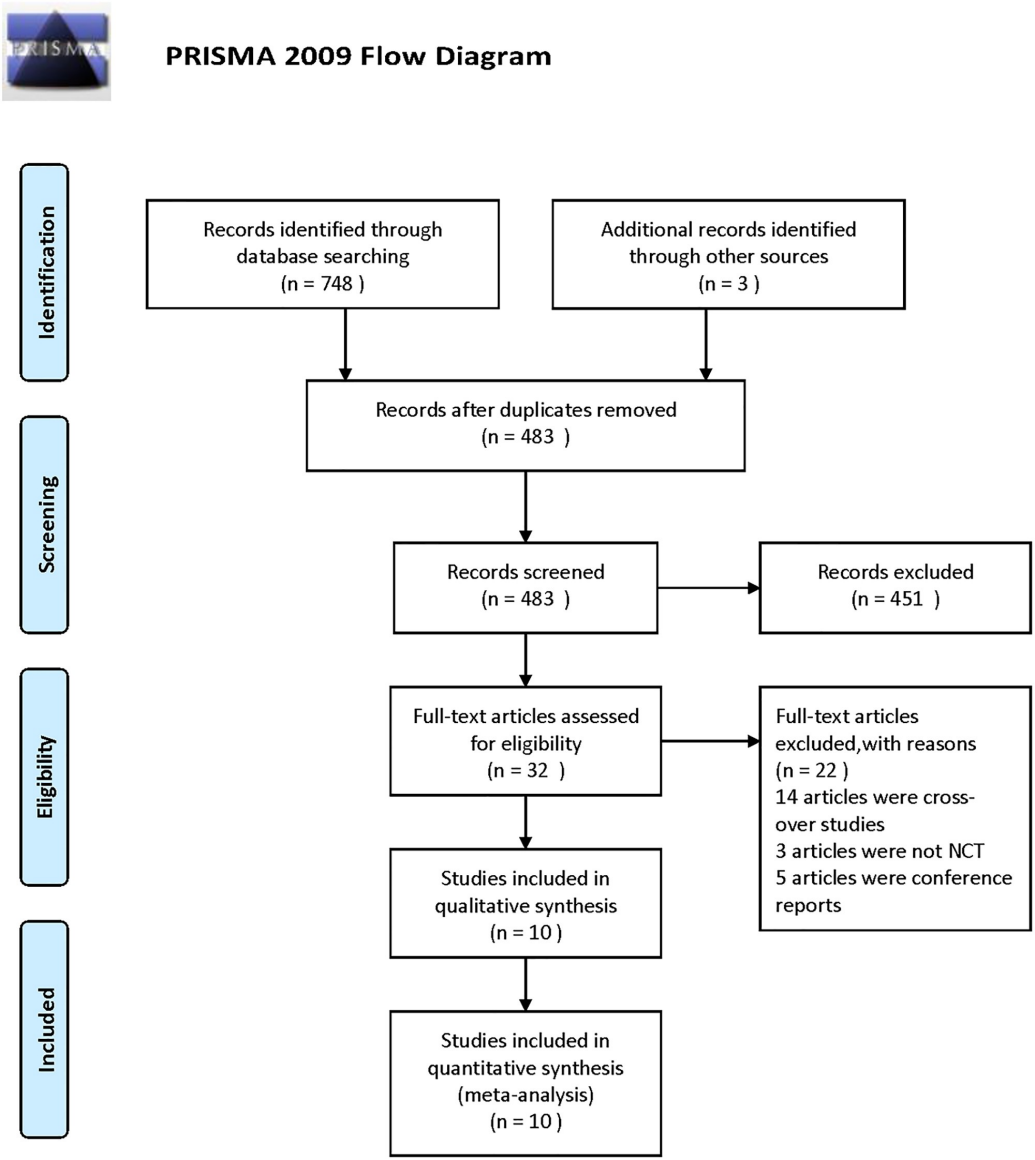
Flow diagram of papers screening. *From*: Moher D, Liberati A, Tetzlaff J, Altman DG, The PRISMA Group (2009). *P*referred *R*eporting *I*tems for *S*ystematic Reviews and *M*eta-*A*nalyses: The PRISMA Statement. PLoS Med 6(7): e1000097. doi: 10.1371/journal.pmed1000097 **For more information, visit** www.prisma-statement.org.

A total of 748 related works of researches were initially retrieved. After many screenings, 10 RCTs were finally included, for a total of 2063 patients. The literature screening process and results are shown in Fig 1. The kappa test showed that the kappa value of agreement during the systematic searches was 0.847.

### 2.2 Basic characteristics of the included studies and bias risk assessment results

**Fig 2:**
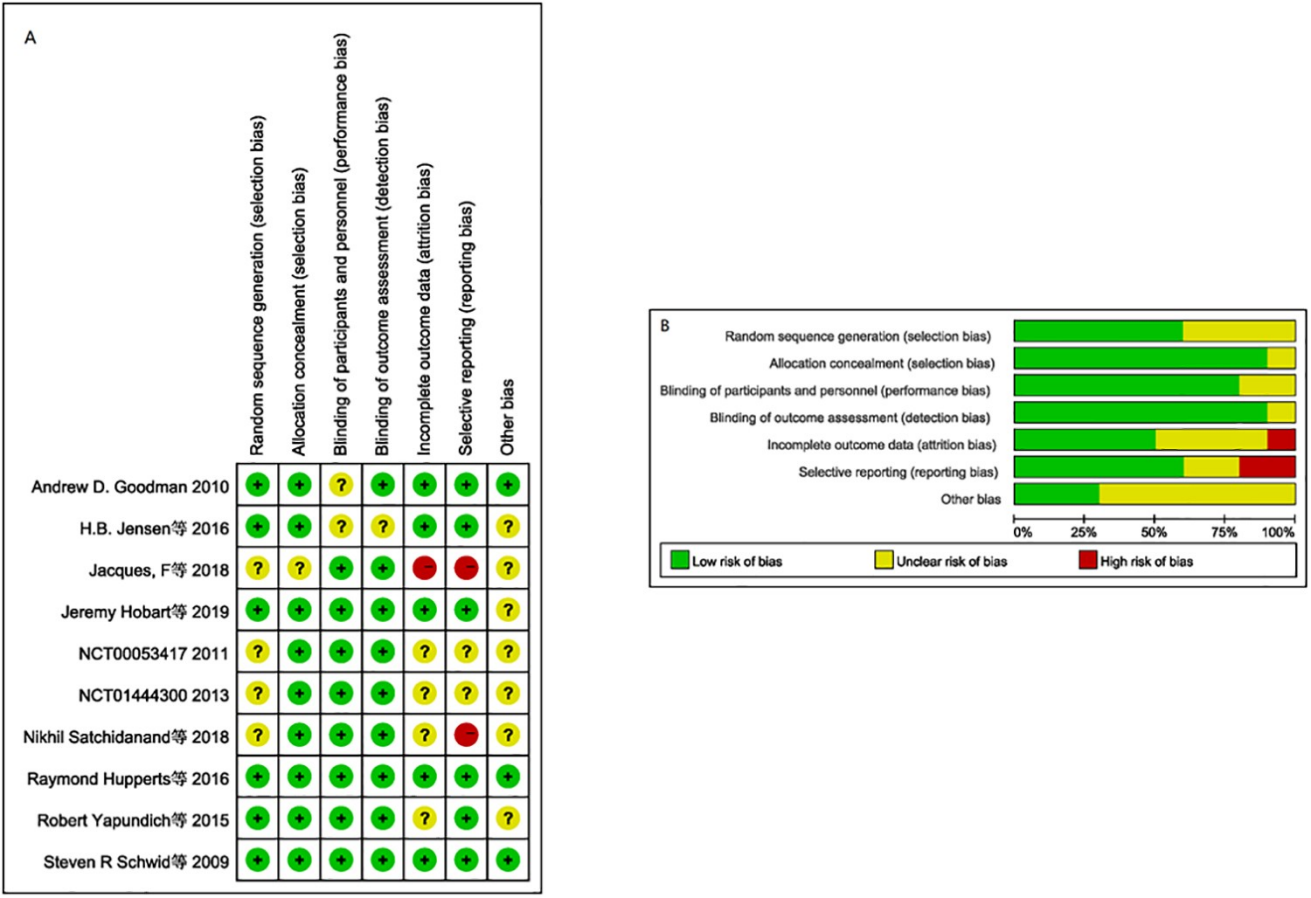
A is the graph of risk of bias summary; B is the graph of risk of bias.

The basic characteristics of the included studies are shown in Table 1. The results of the bias risk assessment are shown in Fig 2. Fig 2A is the graph of risk of bias summary, and Fig 2B is the graph of risk of bias.

**Table 1:** Basic characteristics of the included studies.

Study	Year	Research Type	Intervention	Number of people (n)	Number of women (n)	average age	EDSS	Disease course (year)	Outcome indicator
Jeremy Hobart et al. [14]	2019	Parallel Assignment, RCT	DAP/Placebo	635	366	48.9	4~7	10	②⑤⑥
Nikhil Satchidanand et al. [15]	2018	Parallel Assignment, RCT	DAP/Placebo	61	48	47.6	≤6.5	13.6	①
Jacques, F et al. [16]	2018	Parallel Assignment, RCT	DAP/Placebo	37	26	52.2	4.7	13.7	①③
Raymond Hupperts et al. [17]	2016	Parallel Assignment, RCT	DAP/Placebo	132	71	49.8	4~7	11.6	②⑤⑥
H.B. Jensen et al. [18]	2016	Parallel Assignment, RCT	DAP/Placebo	37	20	49.5	4~7	9.7	④
Robert Yapundich et al. [19]	2015	Parallel Assignment, RCT	DAP/Placebo	429	300	52.6	4.8	12.1	①④⑤⑥
NCT01444300 [20]	2013	Parallel Assignment, RCT	DAP/Placebo	24	11	47.5	NA	12	①⑤
NCT00053417 [21]	2011	Parallel Assignment, RCT	DAP/Placebo	206	131	49.8	NA	15	①⑤⑥
Andrew D. Goodman et al. [22]	2010	Parallel Assignment, RCT	DAP/Placebo	239	162	51.7	1.5~7	13.8	①④⑤⑥
Andrew D Goodman et al. [23]	2009	Parallel Assignment, RCT	DAP/Placebo	300	205	51.4	2.5–7	13.2	①④⑤⑥

^①^ Timed 25-Foot Walk test (T25FW) improvement rate ≥ 20%;

^②^Timed Up and Go test (TUG) improvement rate ≥10%;

^③^Timed 8-meter walk (T8MW) improvement rate ≥20%;

^④^T25FW walking speed change rate;

^⑤^Incidence rate;

^⑥^Urinary infection rate.

### 2.3 Meta-analysis results

#### 2.3.1 Improvement rate

**Fig 3:**
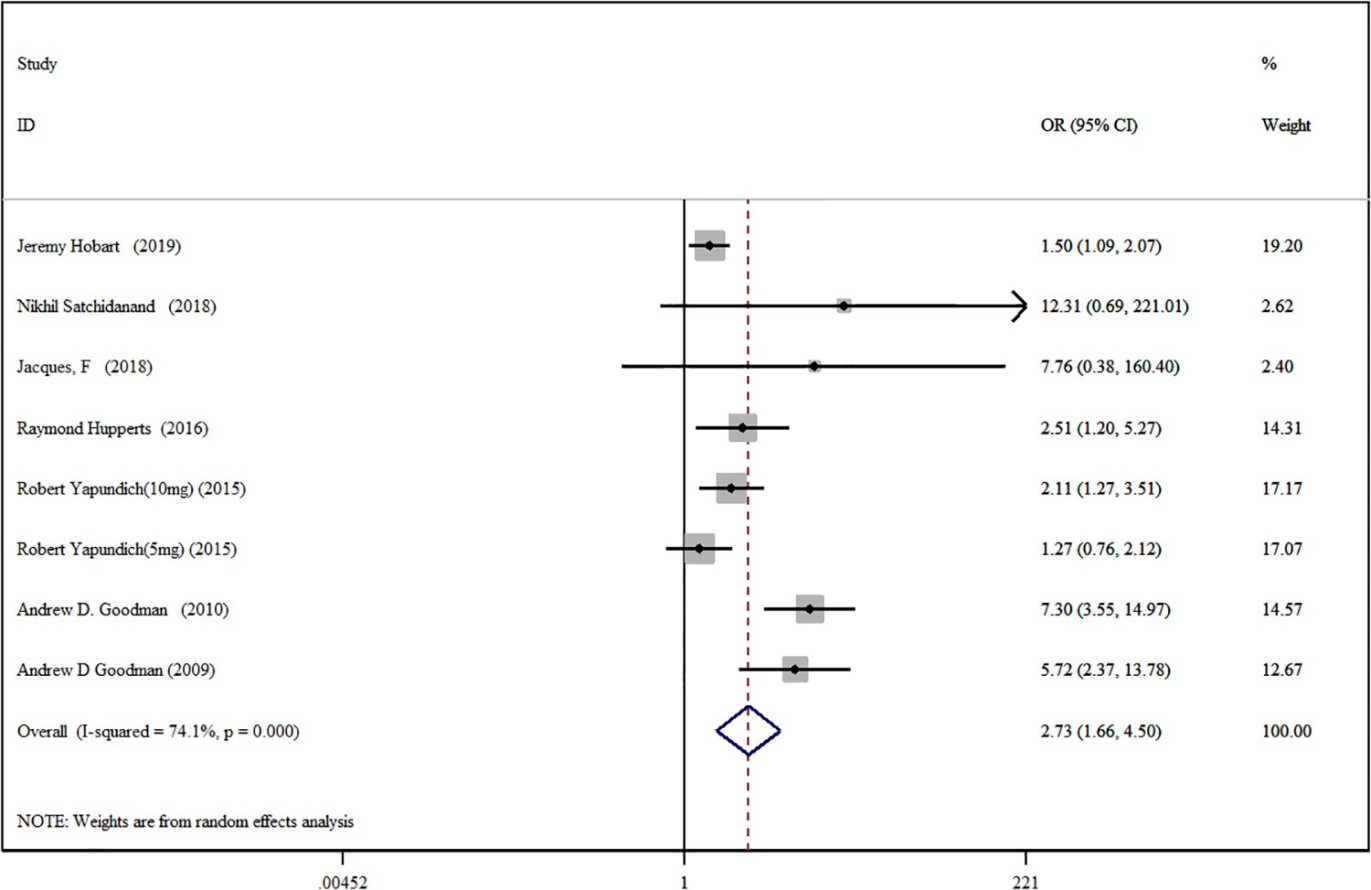
Forest plot of the Mobility Disability improvement rate with DAP and placebo.

There are a total of seven research papers included [14,15,16,17,19,22,23]. Among them, Robert Yapundich et al [19]. used different doses of DAP in comparison with placebo. Accordingly, we divided his study into 2 RCTs, i.e., there are a total of 8 RCTs included in the study. Wherein, the DAP test group has a total of 1071 people, and the placebo control group has 774 people. Random analysis model meta-analysis results show that the DAP test group improved the Mobility Disability significantly better than the placebo control group [OR = 2.73, 95%CI (1.66, 4.50), P<0.001, I^2^ = 74.1%] (Fig 3). After significant heterogeneity between the observed therapeutic effects, we implemented regression analysis on the test methods, study areas, publication years, and doses, and they were not found to have a significant impact on the homogeneity of the included studies (Table 2). This heterogeneity may be due to differences in the expanded disability status scale (EDSS) included in each study, i.e., the degree differences of Mobility Disability among the patients who were included in this research. Egger test did not detect significant publication bias (P = 0.071).

**Table 2:** Results of regression analysis.

logor	Coef.	Std. Err.	t	p>|t|	[95% Conf.	Interval]
year	-0.13064	0.087445	-1.49	0.209	-0.373431	0.112144
method	0.163393	0.646455	0.25	0.813	-1.631454	1.95824
country	0.186961	0.387008	0.48	0.654	-0.887546	1.261467
dosage	-.9098783	.5184995	-1.75	0.178	-2.559975	.7402185
_cons	264.0062	176.4359	1.5	0.209	-225.8585	753.8709

#### 2.3.2 T25FW average walking speed change rate

**Fig 4:**
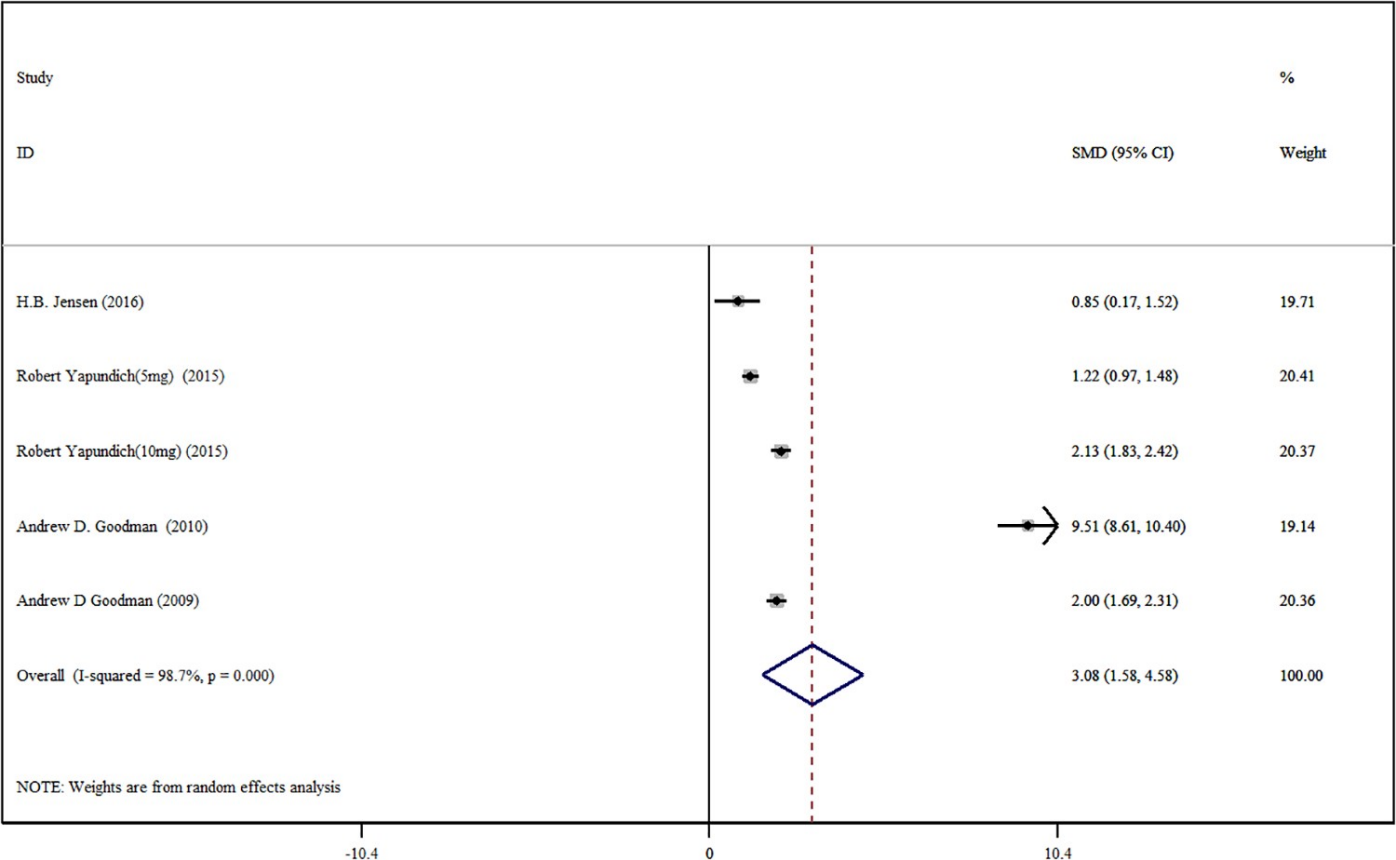
Forest plot of the average walking speed change rate of T25FW with DAP and placebo.

There are a total of 4 research papers included [18,19,22,23]. Among them, Robert Yapundich et al [17]. used different doses of DAP in comparison with placebo. We divided his study into 2 RCTs, i.e., a total of 5 RCTs. There were 644 patients in the DAP test group and 347 patients in the placebo control group. Random analysis model meta-analysis results show that the average rate of change in walking speed in the DAP test group was much higher than the placebo control group [SMD = 3,08, 95%CI(1,58, 4.58), P<0.001, I^2^ = 98.7%] (Fig 4). Egger test does not detect significant publication bias (P = 0.224). Although the result shows that there is some heterogeneity in the meta-analysis, regression analysis about the dosage of DFP (P = 0.980) and publication years (P = 0.577) show that these do not affect the heterogeneity of research cases included. Among them, the differences in personal situations (such as career, degree of Mobility Disability) may the reason for this heterogeneity in the research cases included. Moreover, the measuring result of walking speed from the T25FW has obvious real-world relevance and has correlated well with other measures of walking and lower extremity function [24].

#### 2.3.3 incidence of adverse events

**Fig 5:**
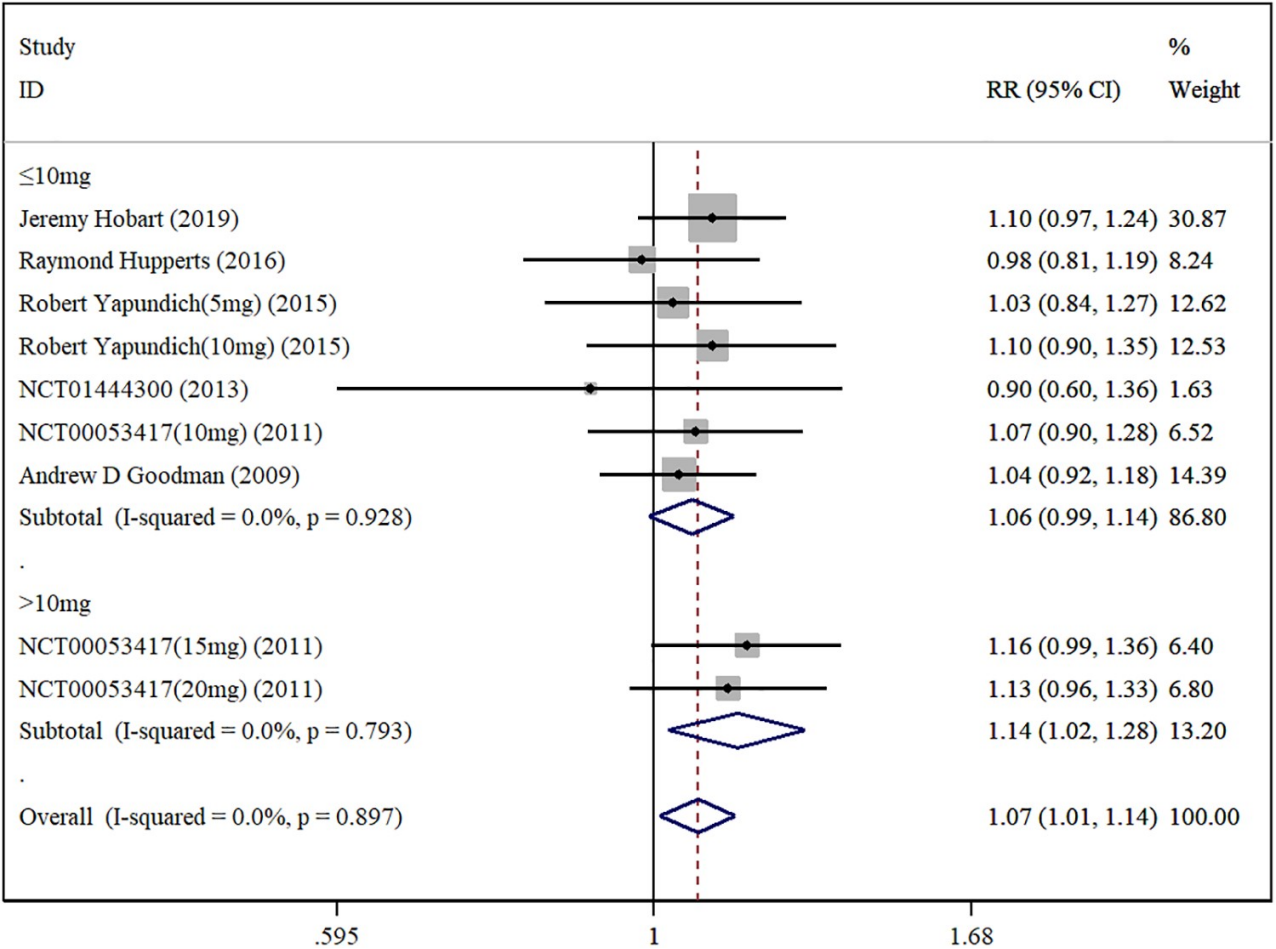
Forest plot of adverse events incidence with DAP and placebo.

There are a total of four research papers and two RCTs included [14,17,19,20,21,23]. Among them, Robert Yapundich et al [19]. and NCT00053417 [21] uses different doses of DAP compared with placebo. We divided them into two RCTs and three RCTs respectively, i.e., there are in total of 9 RCTs in the study. One thousand sixty-nine patients were in the DAP test group and 657 patients in the placebo control group. Fixed-effects model meta-analysis results show that [RR = 1.07, 95%CI(1.01, 1.14), P = 0.897, I^2^ = 0.0%] (Fig 5). The results of subgroup analysis show that there are no significant differences of the adverse effects rate between DAP test group (Doses≤10mg) and placebo control group[RR = 1.06, 95%CI(0.99, 1.14), P = 0.928, I^2^ = 0.0%]; and the adverse effects rate of the DAP test group (Doses>10mg) is higher than that of the placebo control group [RR = 1.14, 95%C I(1.02, 1.28), P = 0.793, I^2^ = 0.0%]. The corresponding Egger test does not detect publication bias (P = 0.297).

#### 2.3.4 incidence of urinary tract infection

**Fig 6:**
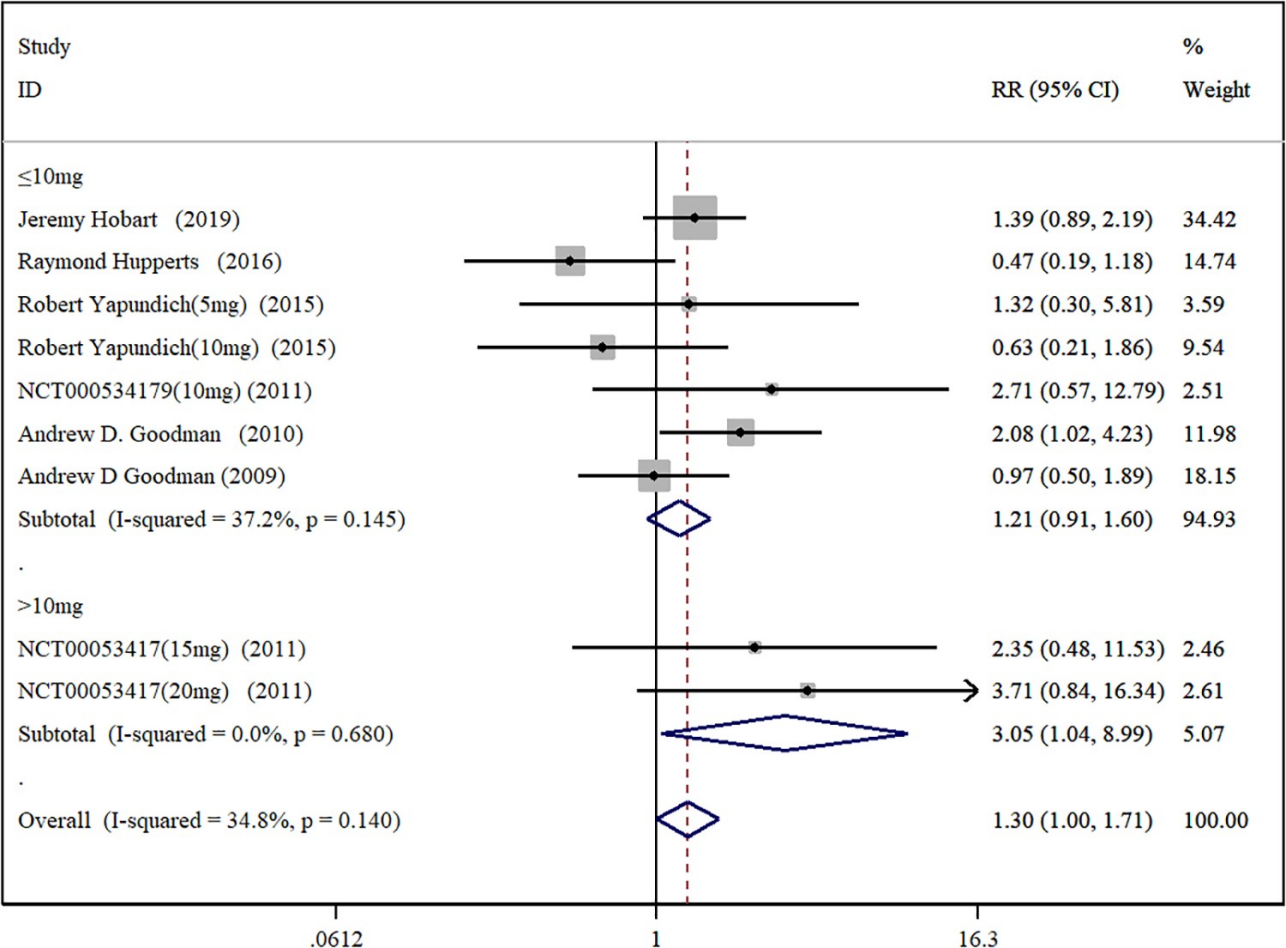
Forest plot of the incidence of urinary tract infection with DAP and placebo.

There are a total of five research papers and one RCTs included [14,17,19,21,22,23]. Among them, Robert Yapundich et al [19]. and NCT00053417 [21] used different doses of DAP compared with placebo. We divided them into two RCTs and three RCTs respectively, i.e., there are in total of 9 RCTs in the study. There were 1177 patients in the DAP test group and 764 patients in the placebo control group. Random analysis model meta-analysis results show that [RR = 1.30, 95%CI(1.00, 1.71), P = 0.140, I^2^ = 34.8%] (Fig 6). In summary, there is no significant difference in the incidence of urinary tract infection between the DAP test group and the placebo control group. However, subgroup analysis shows that the incidence of urinary tract infection of the DAP test group (Doses>10mg) is higher than the placebo control group [RR = 3.05, 95%CI(1.04, 8.99), P = 0.680, I^2^ = 0.0%]. The corresponding Egger test does not detect publication bias (P = 0.731).

### 2.4 Publication bias and sensitivity analysis

**Fig 7:**
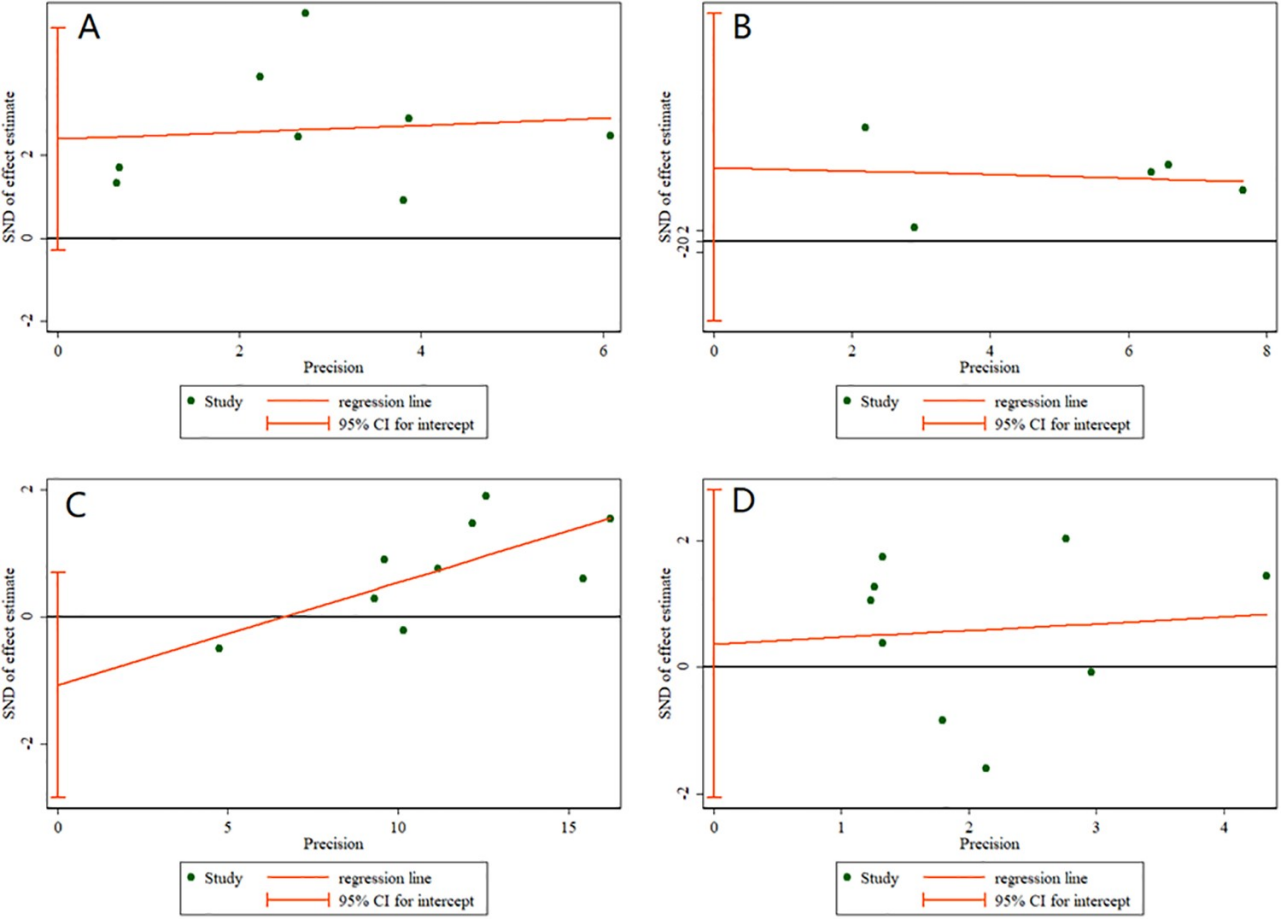
A is the Egger plot of Mobility Disability improvement rate with DAP and placebo. B is the Egger plot of average walking speed change rate of T25FW with DAP and placebo. C is the Egger plot of adverse events incidence with DAP and placebo. D is the Egger plot of incidence of urinary tract infection with DAP and placebo.

In order to ensure the influences of an individual trial to the research results, we analyzed the sensitivity across the included studies(S1 Fig). We did not discover any significant impact from any single study and confirmed the direction of the results. Combined with egger test (Fig 7), we used Funnel Plots to analysis the RCTs which the sample size is more significant than five (S2 Fig), and the results show that there is no significant publication bias of all variables in our meta-analysis.

## 3 Discussion

Mobility Disability is a chronic inflammatory disease that acts on the central nervous system. More than 75% of Mobility Disability patients experience varying degrees of walking disability and decreased mobility after 17 years of onset [25]. Although the incidence of Mobility Disability in Mobility Disability patients is high, there are few drugs to treat it. DAP is the only drug approved by the US FDA to improve the Mobility Disability of the disease [26]. DAP significantly and continuously improves the walking ability of some patients with Multiple Sclerosis by blocking the potassium channels of demyelinated axons and suppressing neural signaling [9–10]. This meta-analysis was the first one to systematically evaluate the safety and efficacy of DAP in the treatment of Multiple Sclerosis Mobility Disability in order to bring hope to patients with Multiple Sclerosis Mobility Disability.

The meta-analysis included 10 RCTs, and the total number of patients was 2,100. The analysis showed that DAP could improve Mobility Disability in patients effectively with Mobility Disability and promote the walking speed compared with placebo. Nonetheless, the research of Robert Yapundich et al [19]. shows that the improvement effect of Mobility Disability is different when patients take different doses of DAP. Among them, the Mobility Disability of the patients, who take DAP twice a day, once 10 mg, will be improved significantly. On the contrary, the improvement of Mobility Disability, which the patients take DAP twice a day, once 5 mg, is not significant.

Concerning the incidence of the adverse events, our meta-analysis has agreement conclusion with the review of Cornblath et al [27]. The incidence of the adverse events of patients who take DAP is slightly higher than that in the place control group. The mechanism of action of DAP on human bodies is the potential explanations for this phenomenon, which is related to the stimulation of DAP to the nervous system.[10] In detail, potassium channel blockade can excite the excitatory state of neuronal and amplify synaptic transmission throughout the brain and spinal cord to result in balance problems, paresthesias, dizziness, anxiety, insomnia, and confusion. The results of the subgroup analysis show that the incidence of adverse events is only increased when patients take DAP once bigger than 10 mg, and some research cases [28] also proved that. Although our analysis confirmed the relationship of the doses of DAP and the incidence of adverse events, we need more high-quality RCTs to confirm this conclusion due to the few numbers of the correlation publications (only two RCTs).

MS will bring various pathological change to the central nervous system, which can lead urinary bladder to dysfunction. Therefore, Mobility Disability patients are more likely than ordinary being to occur urinary system infection, and any additional risks of urinary system infection will cause clinical attentions. This meta-analysis also reported the incidence rate of urinary system infection and results show that there are no significant differences between DAP test group and the placebo control group of the incidence rate of urinary system infection, which is disagreement with the conclusion of the review of Cornblath et al. Among them, DAP will gather together in urine to produce high concentration DAP urine after the metabolism process in kidney because DAP do not participate the metabolism process in kidney, as well as most urinary system infection cases are confirmed according to clinical symptoms but not direct diagnosis. Thus, some symptoms in mild or moderate urinary tract infection may derive from the stimulation of the high concentration DAP urine to urethral canal or/and direct excited of motor nerve ending. It could explain the differences in incidence between the review of Cornblath et al. and our meta-analysis. Meanwhile, subgroup analysis shows that the incidence rate of urinary system infection will increase when patients take more DAP doses. However, to verify this conclusion, we need more research cases because the research cases, which included in the meta-analysis, mostly used the data of patients who took DAP once 10 mg.

## 4 Limitations

This meta-analysis has some particular limitations which are mainly in the following aspects: 1) This meta-analysis only included English literature and may miss some studies in other languages. 2) Some heterogeneity in part of the research may come from the difference in degree in Expanded Disability Status Scale. 3) Egger’s publication bias test and results of meta-regression analysis should be treated carefully due to the number of research cases, which included in the meta-analysis, is small. 4) This meta-analysis does not register on PROSPERO, that little bias may exist although we followed the criteria and step of systematic review strictly.

## 5 Conclusions

In summary, DAP, which is safe and reliable when taking it in specific doses, can effectively improve Mobility Disability caused by Multiple Sclerosis patients, and able to increase the walking speed. The meta-analysis did not analyze the aspect of the dosage of DAP in clinical usage due to the limitation of the number of correlation research cases. Therefore, more and higher-quality randomized controlled clinical trials are needed in the future to verify the results of this meta-analysis further.

## Supporting information

S1 Appendix PRISMA 2009 checklist. (DOC)

S1 File Search strategies. (DOCX)

S1 Fig Sensitivity analysis (A: Improvement rate; B: incidence of adverse events; C: incidence of urinary tract infection). (TIF)

S2 Fig Funnel plots (A: incidence of adverse events; B: incidence of urinary tract infection). (TIF)
