# Supplementary material for: Study on Dalfampridine in the treatment of Multiple Sclerosis Mobility Disability: A meta-analysis
Source: PLoS One. 2019 Sep 12;14(9):e0222288. doi: 10.1371/journal.pone.0222288 (PMC6742383; doi:10.1371/journal.pone.0222288)
Supplement: S1 File — (DOCX) [file pone.0222288.s002.docx]

PubMed:

((((((((Sclerosis, Multiple[Title/Abstract]) OR Sclerosis, Disseminated[Title/Abstract]) OR Disseminated Sclerosis[Title/Abstract]) OR MS (Multiple Sclerosis)[Title/Abstract]) OR Multiple Sclerosis, Acute Fulminating[Title/Abstract])) OR "Multiple Sclerosis"[Mesh])) AND ((((((((((((Multiple Sclerosis, Acute Fulminating[Title/Abstract]) OR 4 Aminopyridine[Title/Abstract]) OR Dalfampridine[Title/Abstract]) OR Pymadine[Title/Abstract]) OR VMI-103[Title/Abstract]) OR VMI 103[Title/Abstract]) OR VMI103[Title/Abstract]) OR 4-Aminopyridine Sustained Release[Title/Abstract]) OR 4 Aminopyridine Sustained Release[Title/Abstract]) OR Sustained Release, 4-Aminopyridine[Title/Abstract]) OR Fampridine-SR[Title/Abstract]) OR Fampridine SR[Title/Abstract])

Web of Knowledge:

((Multiple Sclerosis) or (Sclerosis, Multiple) or (Sclerosis, Disseminated) or (Disseminated Sclerosis) or (MS (Multiple Sclerosis)) or (Multiple Sclerosis, Acute Fulminating)) and ((4-Aminopyridine) or (4 Aminopyridine) or (Dalfampridine) or (Pymadine) or (VMI-103) or (VMI 103) or (VMI103) or (4-Aminopyridine Sustained Release) or (4 Aminopyridine Sustained Release) or (Sustained Release, 4-Aminopyridine) or (Fampridine-SR) or (Fampridine SR))

Embase:

#1 ‘Multiple Sclerosis’/exp

#2 ‘Sclerosis, Multiple’:ab,ti

#3 ‘Sclerosis, Disseminated’:ab,ti

#4 ‘Disseminated Sclerosis’:ab,ti

#5 ‘MS (Multiple Sclerosis)’:ab,ti

#6 ‘Multiple Sclerosis, Acute Fulminating’:ab,ti

#7 #1 OR #2 OR #3 OR #4 OR #5 OR #6

#8 ‘4-Aminopyridine’/exp

#9 ‘4 Aminopyridine:ab,ti

#10 ‘Dalfampridine’:ab,ti

#11 ‘Pymadine’:ab,ti

#12 ‘VMI-103’:ab,ti

#13 ‘VMI 103’:ab,ti

#14 ‘VMI103’:ab,ti

#15 ‘4-Aminopyridine Sustained Release’:ab,ti

#16 ‘4 Aminopyridine Sustained Release’:ab,ti

#17 ‘Sustained Release, 4-Aminopyridine’:ab,ti

#18 ‘Fampridine-SR’:ab,ti

#19 ‘Fampridine SR’:ab,ti

#20 #8 OR #9 OR #10 OR #11 OR #12 OR #13 OR #14 OR #15 OR #16 OR #17 OR #18 OR #19

#21 #7 AND #20

Cochrane:

#1 MeSH descriptor:[Multiple Sclerosis] explode all trees

#2 MeSH descriptor:[ 4-Aminopyridine] explode all trees

#3 Multiple*:ti,ab,kw (Word variations have been searched)

#4 Disseminated*:ti,ab,kw (Word variations have been searched)

#5 Sclerosis*:ti,ab,kw (Word variations have been searched)

#6 #3 OR #4

#7 #6 AND #5

#8 #1 OR #7

#9 Aminopyridine*:ti,ab,kw (Word variations have been searched)

#10 Dalfampridine*:ti,ab,kw (Word variations have been searched)

#11 Pymadine*:ti,ab,kw (Word variations have been searched)

#12 Fampridine*:ti,ab,kw (Word variations have been searched)

#13 #9 OR #10 OR #11 OR #12

#14 #2 OR #13

#15 #8 AND #14

ClinicalTrials.gov:

Condition or disease: Multiple Sclerosis

Other terms: Dalfampridine
