# Supplementary figures and images for: Study on Dalfampridine in the treatment of Multiple Sclerosis Mobility Disability: A meta-analysis
Source: PLoS One. 2019 Sep 12;14(9):e0222288. doi: 10.1371/journal.pone.0222288 (PMC6742383; doi:10.1371/journal.pone.0222288)

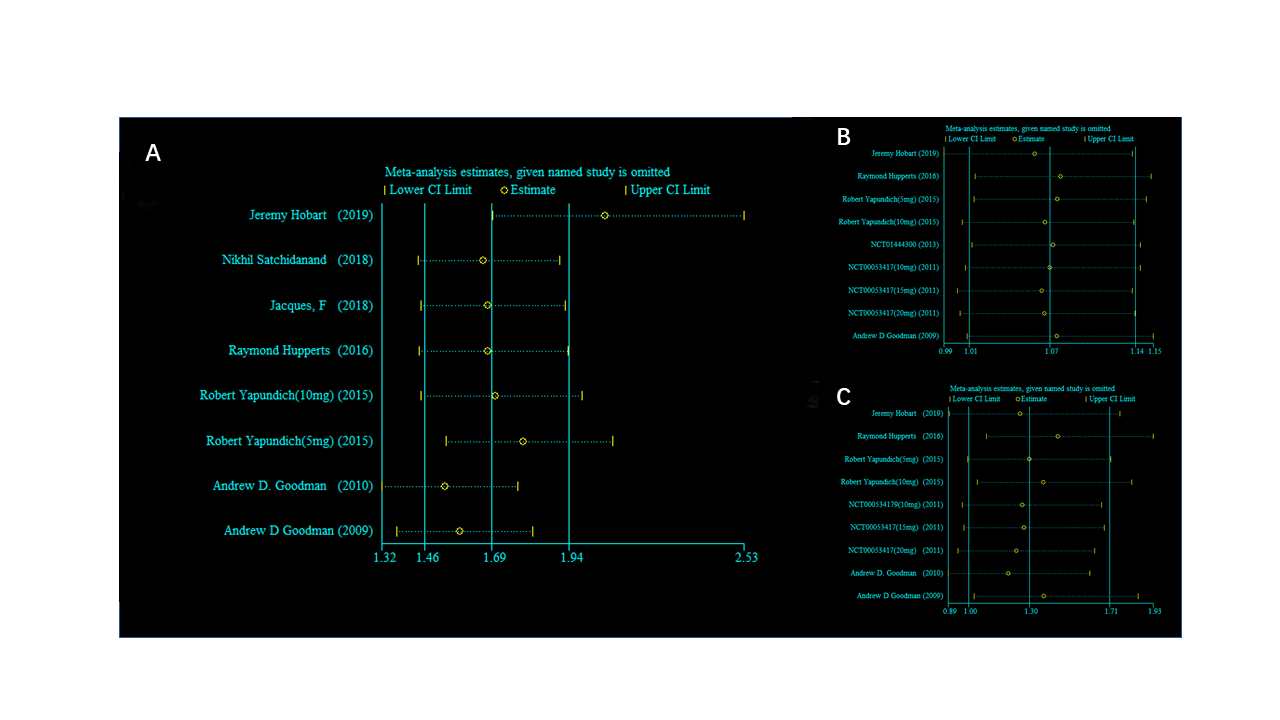

Supplement: S1 Fig — (TIF) [file pone.0222288.s003.tif]

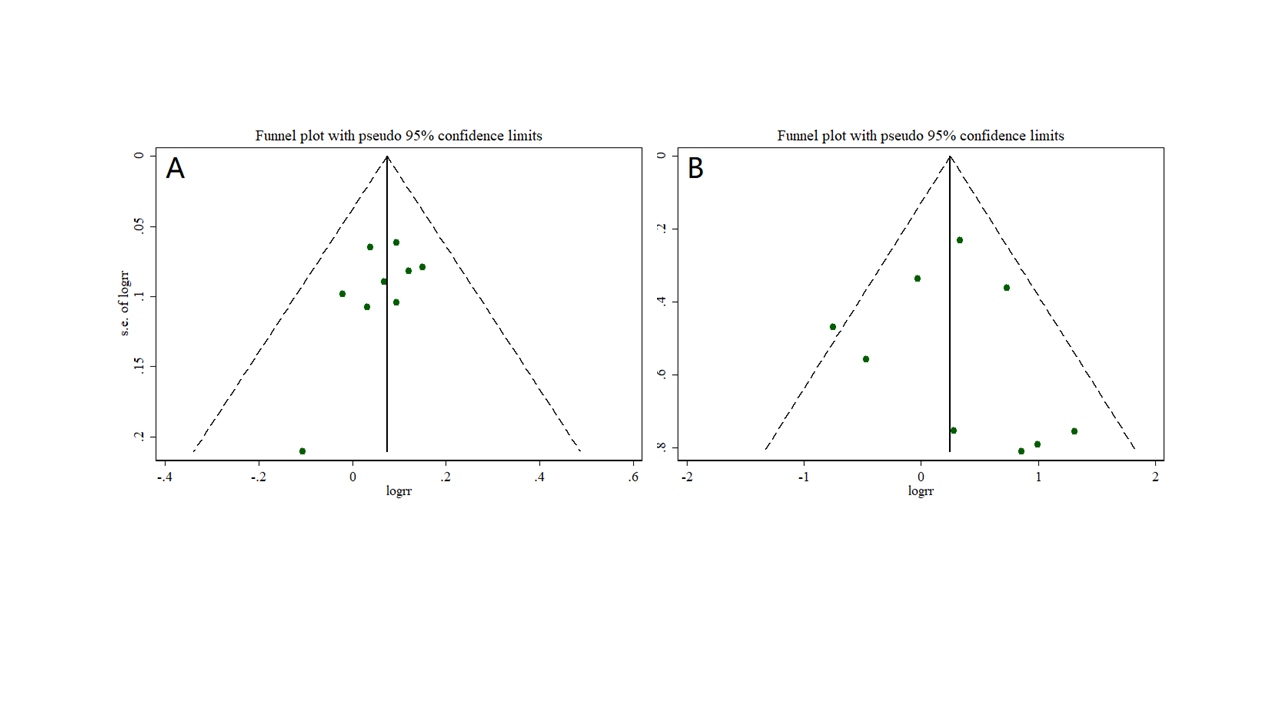

Supplement: S2 Fig — (TIF) [file pone.0222288.s004.tif]
